# A dual decoder U-Net-based model for nuclei instance segmentation in hematoxylin and eosin-stained histological images

**DOI:** 10.3389/fmed.2022.978146

**Published:** 2022-11-11

**Authors:** Amirreza Mahbod, Gerald Schaefer, Georg Dorffner, Sepideh Hatamikia, Rupert Ecker, Isabella Ellinger

**Affiliations:** ^1^Institute for Pathophysiology and Allergy Research, Medical University of Vienna, Vienna, Austria; ^2^Research Center for Medical Image Analysis and Artificial Intelligence, Department of Medicine, Danube Private University, Krems an der Donau, Austria; ^3^Department of Computer Science, Loughborough University, Loughborough, United Kingdom; ^4^Institute of Artificial Intelligence, Medical University of Vienna, Vienna, Austria; ^5^Austrian Center for Medical Innovation and Technology, Wiener Neustadt, Austria; ^6^Department of Research and Development, TissueGnostics GmbH, Vienna, Austria

**Keywords:** digital pathology, medical image analysis, nuclei segmentation, machine learning, deep learning

## Abstract

Even in the era of precision medicine, with various molecular tests based on omics technologies available to improve the diagnosis process, microscopic analysis of images derived from stained tissue sections remains crucial for diagnostic and treatment decisions. Among other cellular features, both nuclei number and shape provide essential diagnostic information. With the advent of digital pathology and emerging computerized methods to analyze the digitized images, nuclei detection, their instance segmentation and classification can be performed automatically. These computerized methods support human experts and allow for faster and more objective image analysis. While methods ranging from conventional image processing techniques to machine learning-based algorithms have been proposed, supervised convolutional neural network (CNN)-based techniques have delivered the best results. In this paper, we propose a CNN-based dual decoder U-Net-based model to perform nuclei instance segmentation in hematoxylin and eosin (H&E)-stained histological images. While the encoder path of the model is developed to perform standard feature extraction, the two decoder heads are designed to predict the foreground and distance maps of all nuclei. The outputs of the two decoder branches are then merged through a watershed algorithm, followed by post-processing refinements to generate the final instance segmentation results. Moreover, to additionally perform nuclei classification, we develop an independent U-Net-based model to classify the nuclei predicted by the dual decoder model. When applied to three publicly available datasets, our method achieves excellent segmentation performance, leading to average panoptic quality values of 50.8%, 51.3%, and 62.1% for the CryoNuSeg, NuInsSeg, and MoNuSAC datasets, respectively. Moreover, our model is the top-ranked method in the MoNuSAC post-challenge leaderboard.

## 1. Introduction

Evaluation of images obtained from tissue sections stained with hematoxylin and eosin (H&E) has long been the gold standard method in medicine for disease diagnosis, cancer grading, and treatment decisions (1). While at some point it was predicted that molecular biology would replace traditional histopathology, even in the era of precision medicine, where an ever-growing list of molecular tests based on omics technologies is available to support precision oncology, microscopic analysis and interpretation of the information contained in H&E-stained tissue sections provides critical information for diagnostic and treatment decisions. It is time- and cost-efficient, and can be applied to small amounts of tissue, while rapid intra-operative tissue analysis based on H&E staining of cryosections remains indispensable to assist surgeons in deciding how to proceed with surgery. H&E-stained histological image analysis also provides valuable information for medical scientists studying the pathophysiology of diseases (2, 3).

Interpretation of H&E-stained images by experts such as pathologists, clinicians, or scientists is however the bottleneck of the common manual analysis as it is time-consuming and prone to inter-observer differences. With the advent of microscopy-based slide scanners that acquire and digitize histological images, computer-aided image analysis systems have been introduced to support human experts and to make the process faster and more objective (4). Computerized methods and in particular deep neural network (DNN)-based algorithms have been shown to be capable of providing diagnostic interpretation with similar accuracy to medical experts (5, 6), while computer-aided analysis can also enable the extraction of quantitative and complex qualitative features that are not recognized by human experts (7).

The nuclei are the most prominent cell organelles. Since they are present in almost all eukaryotic cells, their detection enables cell localization. Various intra- and extra-cellular factors determine the nuclear shape. This results in a physiologic variation of nucleus shapes that can be used to identify sub-populations of cells (8). Moreover, there are significant morphological alterations of nuclei in diseases. Cancer, for example, is known to alter nuclear parameters such as size and shape. These variations are thus an important piece of information contributing to tumor diagnosis and grading (9). Consequently, automated detection, segmentation and in some cases classification of nuclei are important processing steps of computer systems used in histological image analysis in the clinical and scientific context.

Various computer-assisted approaches have been proposed for nuclei instance segmentation, ranging from conventional image processing techniques to classical machine learning and advanced deep learning-based approaches (10–12). Image processing techniques such as adaptive thresholding or watershed segmentaion are still widely used for non-sophisticated images. Open-source software packages, such as ImageJ2 (13) or CellProfiler (14) have in-built image processing engines that can be used for microscopic image analysis, for example, for the segmentation of cell nuclei. However, for tissue samples where the nuclei are close together or even overlap or show considerable differences in intensity, such methods generally do not perform well (15, 16). For more complex images, machine learning, and in particular convolutional neural network (CNN)-based approaches, can be exploited (12). In the medical domain where access to fully annotated dataset is limited, more and more semi-supervised and unsupervised approaches are being used to deal with this issue (17, 18). However, supervised deep learning (DL) and specially CNN-based approaches still deliver the best performances in most cases. Supervised CNN algorithms have shown excellent detection, segmentation and classification performance for a range of medical image modalities such as COVID-19 detection in X-ray images (19), cervical cell classification or pollen grain classification in microscopic images (20–22) or foot ulcer segmentation in clinical images (23). CNN-based techniques for nuclei instance segmentation (and classification) can be broadly classified into two main categories, detection-based methods such as Mask R-CNN (24), and encoder-decoder-based approaches such as the U-Net model and its variants (25–27), while more recently, hybrid approaches have also been proposed to perform nuclei instance segmentation in H&E-stained histological images (28–30). Although these methods have shown significant improvement compared to other non-DL-based approaches, a robust and accurate model for the segmentation of nuclei of multiple cell types in different organs that generalizes well for different datasets is still challenging to achieve.

In this paper, we propose a novel architecture, consisting of one encoder and two decoders, to perform nuclei instance segmentation in H&E-stained histological images. While the encoder performs standard feature extraction, the decoders are designed to predict image foreground and distance maps of all nuclei. To verify robustness and generalisability of our segmentation model, we test it on three publicly available datasets and demonstrate it to achieve excellent instance segmentation performance. Moreover, to perform nuclei classification, we develop an independent U-Net-based model that classifies the objects detected by the dual decoder model. Applied on the CryoNuSeg (31) and NuInsSeg (32) datasets (both datasets for instance segmentation of cell nuclei) and the MoNuSAC dataset (16) (a dataset for instance segmentation and classification of cell nuclei), our method yields average panoptic quality (PQ) scores of 50.8%, 51.3%, and 62.1%, respectively. Furthermore, it is the top ranked entry in the MoNuSAC post-challenge leaderboard^1^.

## 2. Method

**Figure 1:**
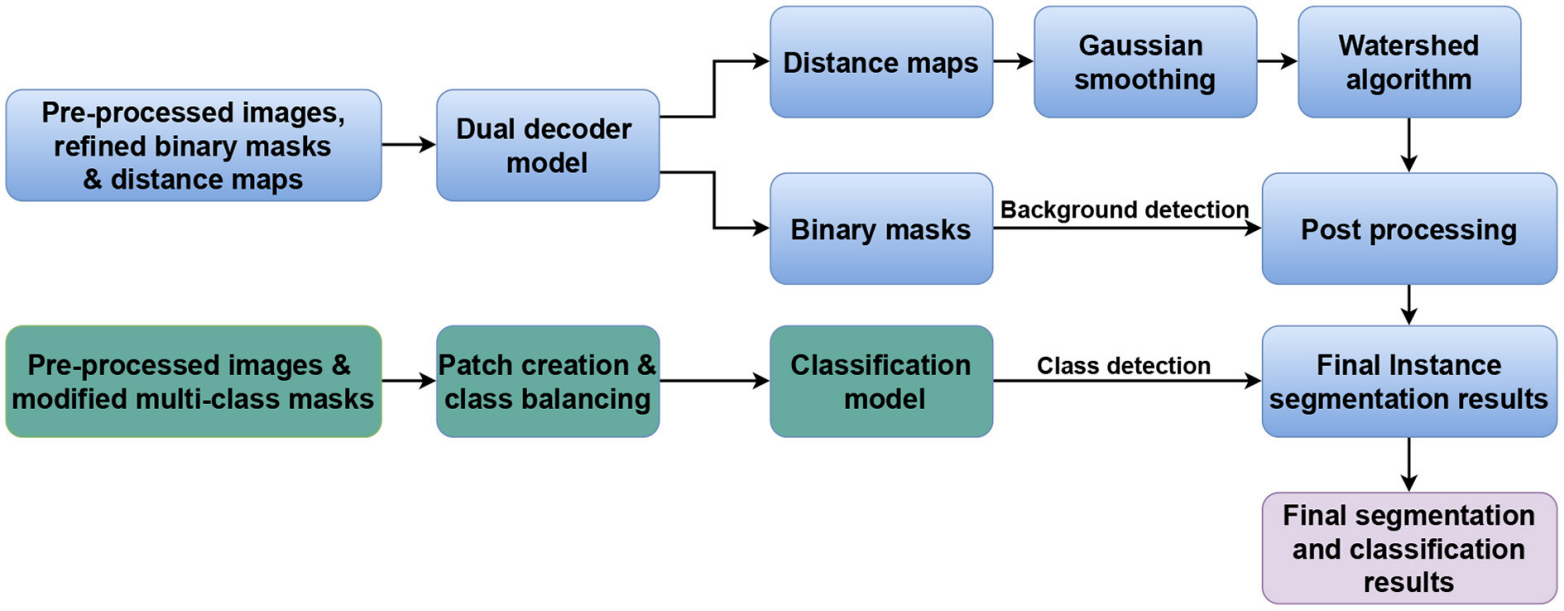
Generic workflow of the proposed method. The blue and green parts represent the nuclei instance segmentation and nuclei classification components, respectively.

Our approach is inspired by our previous work on nuclei instance segmentation in H&E-stained histological images in Mahbod et al. (27). However, in contrast to there, where two separate models were designed to predict nuclei foreground and nuclei distance maps, a single model performs both tasks in our proposed approach. In addition, we also present an independent classification model to extend the workflow to also perform nuclei classification (if required). Figure 1 illustrates the generic workflow of our proposed model for performing nuclei instance segmentation (blue sections) and classification (green sections). In the following, we describe the details of the utilized datasets, our proposed model, and the experimental setup.

### 2.1. Datasets

We use four datasets of H&E-stained histological images, namely the CryoNuSeg (31), NuInsSeg, MoNuSeg (15), and MoNuSAC (16) datasets. Details on how we exploit these datasets in different experiments are given in Section 2.6.

CryoNuSeg, NuInsSeg, and MoNuSeg are manually annotated datasets for nuclei instance segmentation. The CryoNuSeg dataset contains 30 image patches of 512 × 512 pixels from 10 different human organs, NuInSeg comprises 667 image patches of the same size from 31 human and mouse organs, while MoNuSeg contains 44 images of size of 1, 000 × 1, 000 pixels from 9 human organs.

MoNuSAC is a manually annotated dataset for nuclei instance segmentation and classification and has 209 and 101 image patches in the training and test set, respectively. The images are of varying sizes, ranging from 82 × 35 to 1, 422 × 2, 162 pixels, and are derived from four human organs. Four nuclei classes are manually labeled, namely epithelial (21,752 nuclei), lymphocyte (23,460 nuclei), neutrophil (803 nuclei), and macrophage (894 nuclei).

Further details of the datasets can be found in Table 1 and the respective publications/repositories.

**Table 1:** Details of the utilized datasets.

	**# patches**	**# nuclei**	**Magnification**	**# organs**	**Patch size**	**# classes**	**Source**
CryoNuSeg	30	7,596	40 ×	10	512 × 512	-	TCGA
NuInsSeg	665	30,698	40 ×	31	512 × 512	-	IPA
MoNuSeg	44	28,846	40 ×	9	1, 000 × 1, 000	-	TCGA
MoNuSAC	310	46,909	40 ×	4	82 × 35 − 1422 × 2, 162	4	TCGA

TCGA=The Cancer Genome Atlas; IPA= Institute for Pathophysiology and Allergy Research, Medical University of Vienna.

### 2.2. Pre-processing

**Figure 2:**
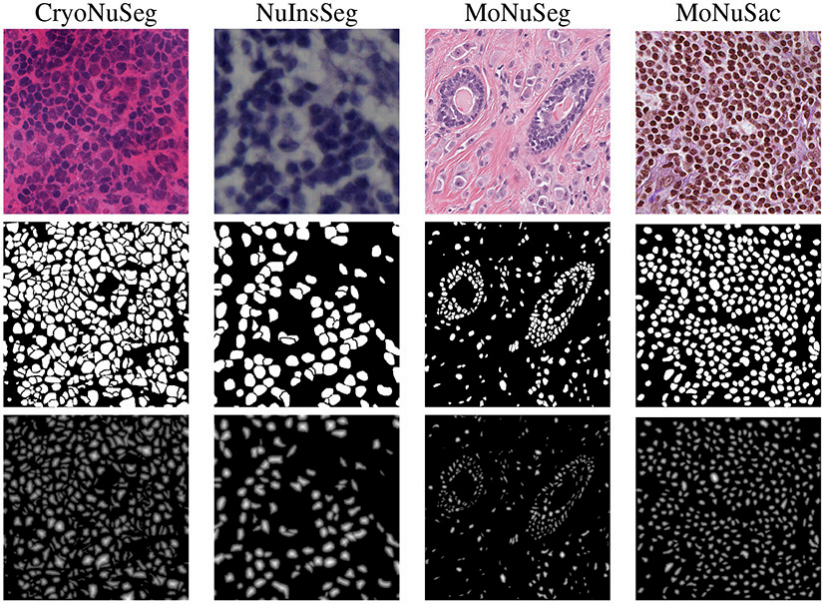
Image examples from the employed datasets (first row), the refined masks generated from ground truth annotations (second row), and the obtained distance maps (third row).

Considering the dataset and the task (either nuclei instance segmentation or nuclei instance segmentation and classification), we apply the following pre-processing steps:

- **Intensity normalization**: we normalize the intensity values of the images in all datasets to the standard range of [0;1] as normalization has shown to be an important step in training a CNN nuclei segmentation model (33).
- **Augmentation**: we apply various forms of morphological and color augmentations during the training phase including random horizontal/vertical flipping, random scaling and random contrast as well as brightness shifts.
- **Generating additional ground truth masks**: we create refined binary masks and elucidation distance maps from the provided manual binary annotations in all datasets to train the dual decoder segmentation model. To generate a refined binary masks, we remove the touching borders between the overlapping nuclei and then apply an erosion operation to obtain a better distinction between close objects as suggested in Mahbod et al. (27). Examples of generated masks for each dataset are shown in Figure 2. For the MoNuSAC training data, we also create multi-class labeled masks to train the classification model. The generated refined binary and labeled masks are only used in the training phase, and for performance evaluation, the originally labeled masks are compared with the model's predictions.

### 2.3. Model

**Figure 3:**
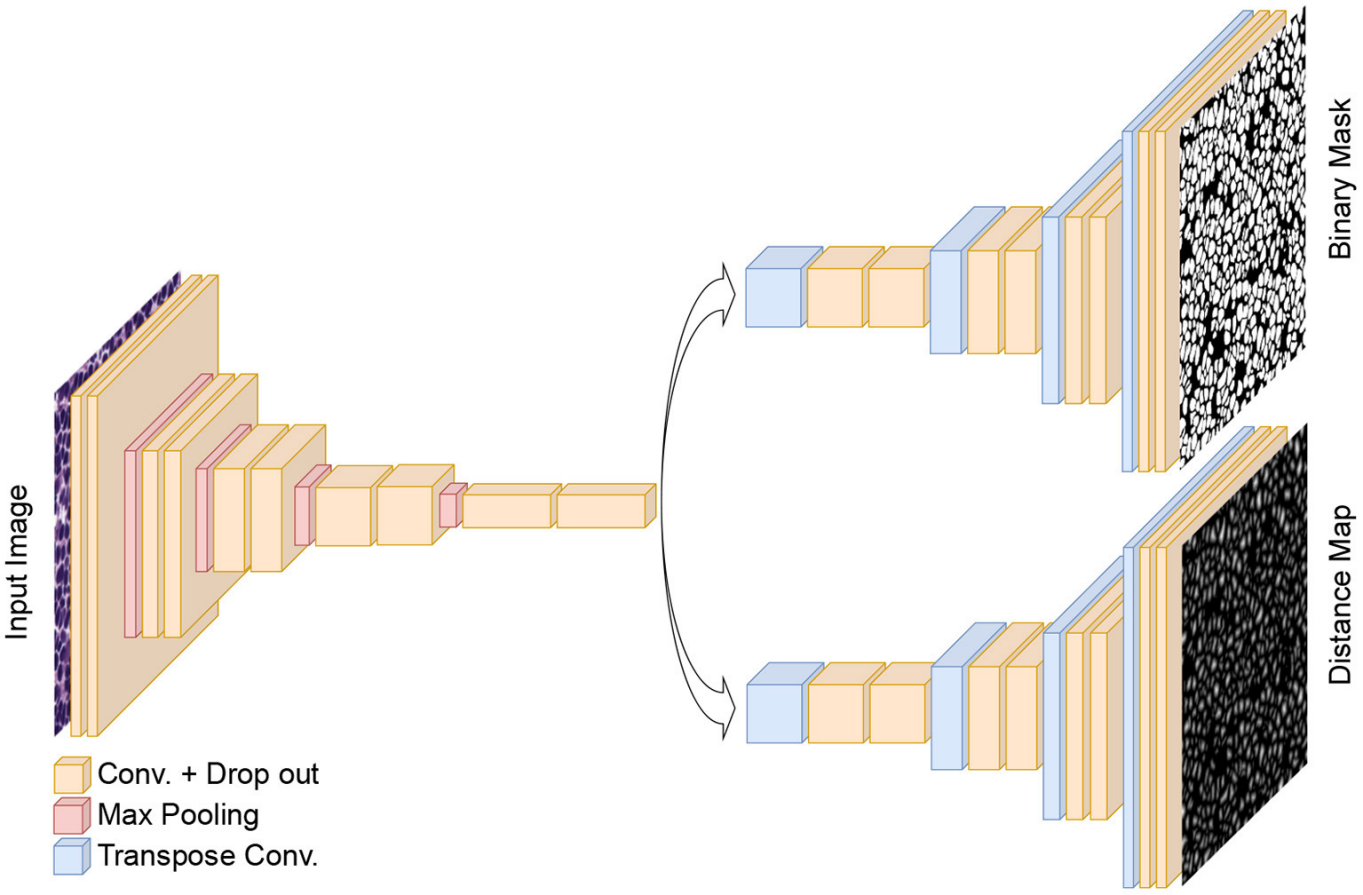
The architecture of the proposed dual decoder model for nuclei instance segmentation. The two decoders are designed to perform binary segmentation and distance map prediction, respectively. For simplicity, skip connections between the encoder and two decoders are not shown.

Our proposed method for nuclei instance segmentation is a dual decoder U-Net-based model. The generic architecture of the developed model is shown in Figure 3. The encoder part of the model has a similar architecture as the original U-Net model, with five convolution blocks, followed by max-pooling layers to extract deep features from the images. In contrast to the original U-Net architecture, we also add drop-out layers between convolutional layers as regularisers (with a rate of 0.1). The generated features in the encoder are then fed to the two decoder paths to predict nuclei foreground and nuclei elucidation distance maps, respectively. The architectures of these two decoders are identical except for the last layer. Both have five convolutional layers, which are equipped with drop-out layers similar to the encoder, and we use transposed convolutional layers in the decoders to up-sample the feature maps. The last activation functions in the first (distance map) and second (binary mask) decoders are linear and sigmoid activations, respectively. We use 3 × 3 convolutional kernels and ReLu activation layers in all other layers, both for encoder and decoder. The loss function of the distance map head is a mean squared error loss function, while the loss function of the binary mask head is a combination of Dice loss and binary cross-entropy loss. We merge the three losses, giving equal weight to each loss term. We utilize the Adam optimiser (34) and an initial learning rate of 0.001 to train the dual decoder model. We train the model for 120 epochs while dropping the learning rate by a factor of 0.1 after every 20 epochs. The model is trained from scratch after Xavier initialization (35) of the weights.

To obtain the final instance segmentation results, the outputs of the dual decoder models are merged as shown in Figure 1. We first calculate the average nucleus size from the semantic segmentation results (binary mask head), and then apply a Gaussian smoothing filter to the distance map predictions with the kernel size of the file derived from the average nucleus size. Finally, we identify the local maxima from the filtered predicted distance maps and use them as seed points for a marker-controlled watershed algorithm (36) to produce the labeled segmented masks.

**Figure 4:**
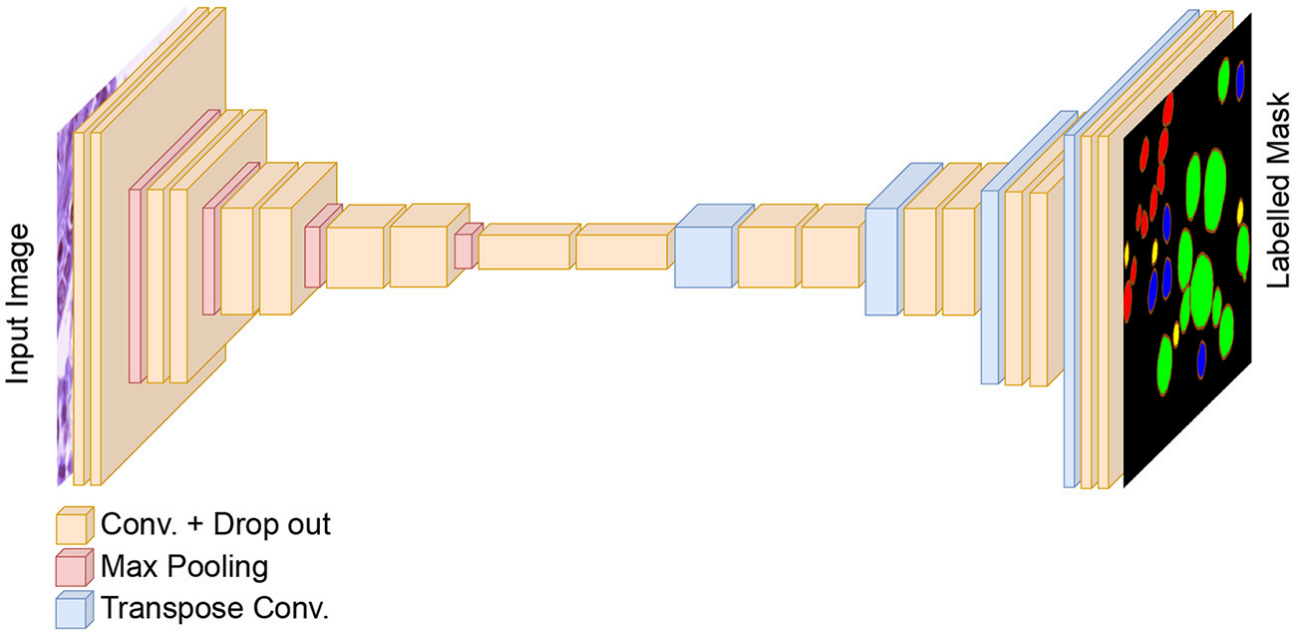
The architecture of the classification model for nuclei instance categorization. The different colors in the output represent the nuclei classes in the MoNuSAC dataset. For simplicity, skip connections between the encoder and two decoders are not shown.

To perform nuclei classification as required in the MoNuSAC challenge, we design an independent U-Net-based classification model to the workflow. The generic architecture of the developed classification model is shown in Figure 4. The encoder and decoder of the classification model are similar to the dual decoder model but with a unique decoder with a softmax activation in the last layer. Moreover, in contrast to the dual decoder model, here we generate four output masks, one for each nucleus class. We use a combination of categorical cross-entropy and Dice loss (with equal weights) as loss function, while the other parameters are identical to the dual decoder model. The output from the classification network is used to determine the nuclei classes of the predicted objects by the dual decoder model. We use a majority voting approach based on the output of the classification model to choose the nucleus type for each object.

### 2.4. Post-processing

We remove tiny detected objects (with an area less than 30 pixels) from the segmentation masks during post-processing. Any holes inside detected nuclei are filled using morphological operations.

For the MoNuSAC experiments, we also remove the vague areas from the final instance segmentation and classification masks. The challenge organizers provide these vague areas for the entire test set images. We use a five-fold cross-validation model ensemble and test-time augmentation (TTA) for our finalized submission for the MoNuSAC post-challenge phase, as these methods have been shown to boost the segmentation performances in other studies (37) including our own (38). We use 90-degree rotated and horizontally flipped images for TTA.

### 2.5. Evaluation

To evaluate the results for the nuclei instance segmentation tasks (CryoNuSeg and NuInsSeg), we use Dice score, aggregate Jaccard Index (AJI), and the panoptic quality (PQ) score. While the Dice score characterizes the general semantic segmentation performance, AJI and PQ score also evaluate a model's ability to separate touching objects and thus quantify instance segmentation performance. A higher Dice score, higher AJI and higher PQ score indicate better performance; further details about the evaluation indices can be found in Graham et al. (39) and Kirillov et al. (40). We also perform statistical Wilcoxon signed-rank test method (37, 41) for each of the evaluation index to compare our model with other approaches.

For the combined nuclei instance segmentation and classification task (MoNuSAC), we use the average PQ score per nuclei class for evaluation. The MoNuSAC challenge organizers performed the evaluation based on the 101 test images of the challenge dataset. Further details about the submission process and multi-class mask format for evaluation can be found on the challenge website^2^ and in Verma et al. (16).

### 2.6. Experimental setup

We conduct three experiments to evaluate the performance of our proposed method. In the first two experiments, we use the CryoNuSeg and NuInsSeg datasets, respectively, to evaluate nuclei instance segmentation performance. In the third experiment, we assess nuclei instance segmentation and classification performance using the MoNuSAC and MoNuSeg datasets with the MoNuSeg dataset only being used for training but not for evaluation purposes. We run our experiments with an identical setup to the one proposed in the reference studies (16, 31) to compare our results with other state-of-the-art algorithms.

For the CryoNuSeg experiment, we follow the 10-fold cross-validation (10CV) scheme proposed in the original study (31), for which the dataset (30 images) is divided into 10-folds (each containing three images) based on the organs. Then, in each CV fold, the images from nine organs are used for training, while the images from the remaining organ are used for testing. We use full-sized images of 512 × 512 pixels both for training and testing.

For the NuInsSeg experiment, we use a 5-fold cross-validation scheme as suggested in the NuInsSeg repository^3^. Full-sized images of 512 × 512 pixels are used for training and testing. We utilize an identical suggested random state to generate the folds.

For the MoNuSAC experiment, we use images of size 256 × 256 randomly cropped from the MoNuSeg dataset to pre-train the dual decoder model. Then, we utilize 256 × 256 cropped images from the MoNuSAC training set to fine-tune the model. Since some MoNuSAC images are smaller than 256 × 256 pixels, we use white pixel padding to create 256 × 256 pixel images. To train the classification model, we extract overlapping patches from the MoNuSAC training images. To address the class imbalance in the dataset, we extract more patches from the underrepresented classes, taking into account the number of nuclei in each class in the training set. In total, 14,862 patches are generated to train the classification model. To evaluate the performance, we use the test set of the MoNuSAC challenge. The test images are first padded (white pixel padding) to create square images and then resized to suitable image sizes (the closest size divisible by 32). We apply the inverse steps to the predicted results to have the final segmentation masks identical to the original MoNuSAC test image sizes. It should be noted that the evaluation in this experiment was performed directly by the challenge organizers.

All experiments are performed on a single workstation with an Intel Core i7-8700 3.20 GHz CPU, 32 GB of RAM and a TITIAN V NVIDIA GPU card with 12 GB of installed memory. Matlab software (version 2020a) is used to prepare the datasets and generate segmentation masks, while the Tensorflow (version 2.4) and Keras (version 2.4) deep learning frameworks are used for model training and testing.

## 3. Results and discussion

The nuclei instance segmentation results on the CryoNuSeg dataset are given in Table 2, which lists the Dice score, AJI and PQ score of our proposed model as well as of several other approaches. The comparative results are split into three sections.

**Table 2:** Nuclei instance segmentation results on CryoNuSeg dataset based on 10CV configuration from Mahbod et al. (31).

	**Dice [%)]**	**AJI [%)]**	**PQ [%)]**
Standard image processing	71.9 (*)	39.9 (*)	32.0 (*)
U-Net+Watershed (25, 36)	79.3 (*)	47.8 (*)	40.4 (*)
Distance U-Net+Watershed (27, 42)	74.7 (*)	48.6(*)	37.5 (*)
Two-stage U-Net (27)	80.3 (*)	52.5 (*)	47.7 (*)
Attention U-Net (43)	79.4 (*)	48.2 (*)	41.7 (*)
Residual attention U-Net (43, 44)	79.8 (*)	49.1 (*)	42.7 (*)
CellPose (45)	77.6 (*)	52.6	50.9
Proposed dual decoder U-Net	81.5	54.1	50.8

(*) signs for each evaluation index show statistical differences (*p* < 0.05) between our proposed method and other approaches.

The first section (first row) compares our method (row 8) with a standard image processing technique using the StrataQuest (SQ) software (version 7.1) ^4^. We use SQ's pre-built image processing engines to derive the results. We use adaptive thresholding, local maxima detection, Watershed algorithm and morphological operations to derive the results. The results show that our model delivers a much better performance considering all three evaluation indices.

The second section (rows 2–4) is an ablation study. This section shows the performance of a single semantic segmentation U-Net (row 2), a single distance U-Net (row 3), and two independent models for semantic segmentation and distance map prediction (row 4) as suggested in Mahbod et al. (27). The results of the ablation study confirm the superior performance of our proposed dual decoder approach (row 8) compared to the sub-models for all three evaluation indices.

The third section (rows 5–7) compares the performance of our method (row 8) with other state-of-the-art DL-based algorithms. As is evident from the ablation study and reported results in the table, our proposed dual decoder U-Net-based model outperforms the other approaches based on the Dice score and AJI and delivers very competitive performance based on the PQ score.

**Figure 5:**
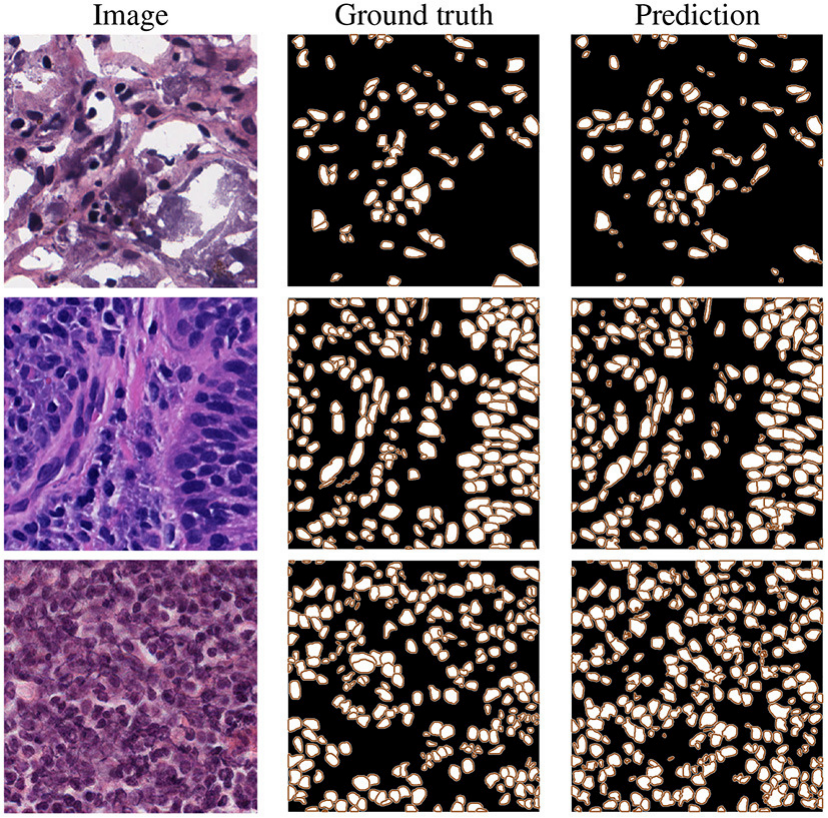
Example results on CryoNuSeg test images, selected from adrenal gland **(top)**, larynx **(middle)**, and lymph node **(bottom)** samples.

It should be noted that for all reported results in the table (besides the standard image processing technique where a set of fixed empirically-driven parameters are used), we utilize the exact same 10CV folds suggested in Mahbod et al. (31). These results confirm our proposed algorithms' excellent semantic and instance segmentation performances. Examples of this performance are given in Figure 5, which shows nuclei instance segmentation results for some CryoNuSeg images.

We report the results of instance segmentation of our model and several others on the NuInsSeg dataset in Table 3, all based on identical 5CV folds as defined in the repository. Similar to the previous experiments for the CryoNuSeg dataset, we report the results in three sections for comparison to standard image processing technique (first row), ablation study (rows 2-4) and comparison to other deep learning- based approaches (rows 5-7). The results in the first two sections confirm the superior performance of our proposed method compared to standard image processing and sub-models derived from our dual decoder architecture.

**Table 3:** Nuclei instance segmentation results on NuInsSeg dataset based on the 5CV configuration from the repository.

	**Dice [%)]**	**AJI [%)]**	**PQ [%)]**
Standard Image processing	47.8 (*)	23.6 (*)	10.7 (*)
U-Net+Watershed (25, 36)	78.8	50.5 (*)	42.8 (*)
Distance U-Net+Watershed (27, 42)	74.1 (*)	50.3 (*)	41.0 (*)
Two-stage U-Net (27)	76.6 (*)	52.7 (*)	47.2 (*)
Attention U-Net (43)	80.5 (*)	45.7 (*)	36.4 (*)
Residual attention U-Net (43, 44)	81.4 (*)	46.2 (*)	36.9 (*)
CellPose (45)	74.7 (*)	52.8 (*)	48.0 (*)
Proposed dual decoder U-Net	79.4	55.9	51.3

(*) signs for each evaluation index show statistical differences (*p* < 0.05) between our proposed method and other approaches.

**Figure 6:**
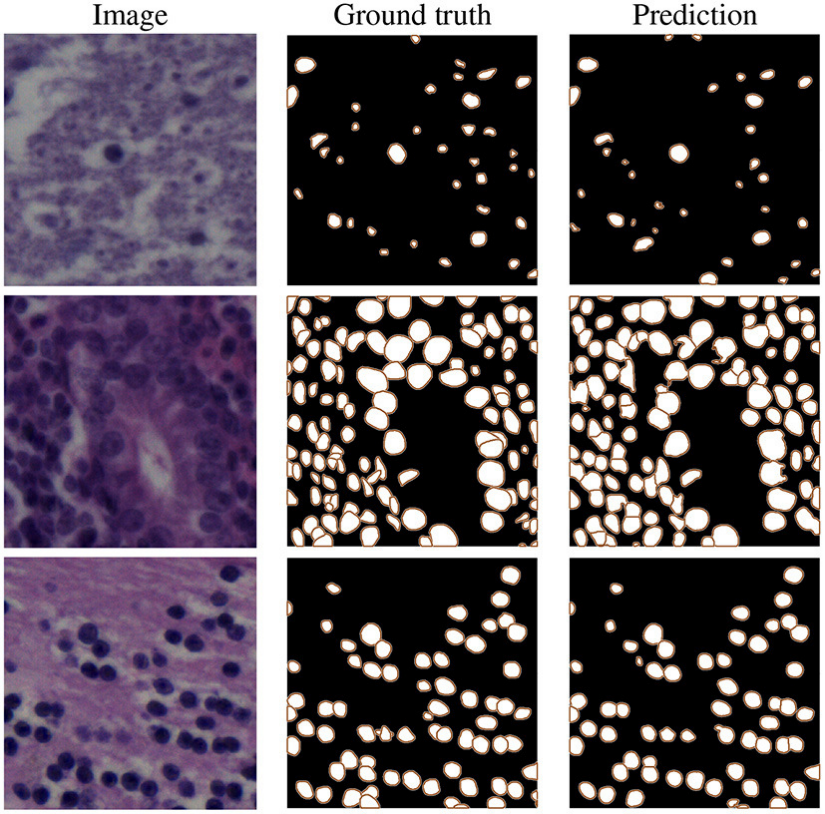
Example results on NuInsSeg test images, selected from human brain **(top)**, human cardia **(middle)**, and human cerebellum **(bottom)** samples.

As we can see in the third section, our proposed model (row 8) clearly achieves the best instance-based segmentation performance (i.e., the highest AJI and PQ score), while delivering slighlty inferior semantic segmentation performance based on the Dice score. In Figure 6, we show some examples of the automatic segmentations obtained from our approach.

The results in Tables 2, 3 also show that the majority of the DL-based results are superior in comparison to the applied standard image processing approach by a large margin, especially in Table 3 (minimum difference of 26.3%, 22.1%, and 25.7% for the Dice, AJI and PQ score, respectively).

The results obtained on the MoNuSAC dataset are given in Table 4. Since the results are directly provided by the challenge organizers and they only report results in terms of average PQ scores, we do so also in the table. It should be noted that the results are slightly different from the original report in Verma et al. (16) since the authors of Verma et al. (16) detected a bug in the evaluation code; the official updated results (identical to those in Table 4) are available in Verma et al. (46), while further details about the evaluation error can be found in Foucart et al. (47).

**Table 4:** Nuclei instance segmentation and classification results on the MoNuSAC challenge test data in terms of average PQ scores for different nucleus classes.

**Team**	**Epithilial cells**	**Lymphocytes**	**Macrophages**	**Neutrophils**	**Average**	**Rank**
TIA-Lab	60.3	63.5	63.1	66.5	65.8	L1
SJTU-426	62.2	56.0	61.2	63.0	61.8	L2
IIAI	60.1	55.6	60.5	61.3	60.5	PL2
Sharif_hooshpardaz	55.2	54.5	50.2	60.0	58.2	PL3
IVG	56.7	45.8	51.2	60.0	55.3	L3
Proposed	61.0	57.1	55.4	65.2	62.1	PL1

In the rank column, L represents the legacy leaderboard, while PL refers to the post-challenge leaderboard.

**Figure 7:**
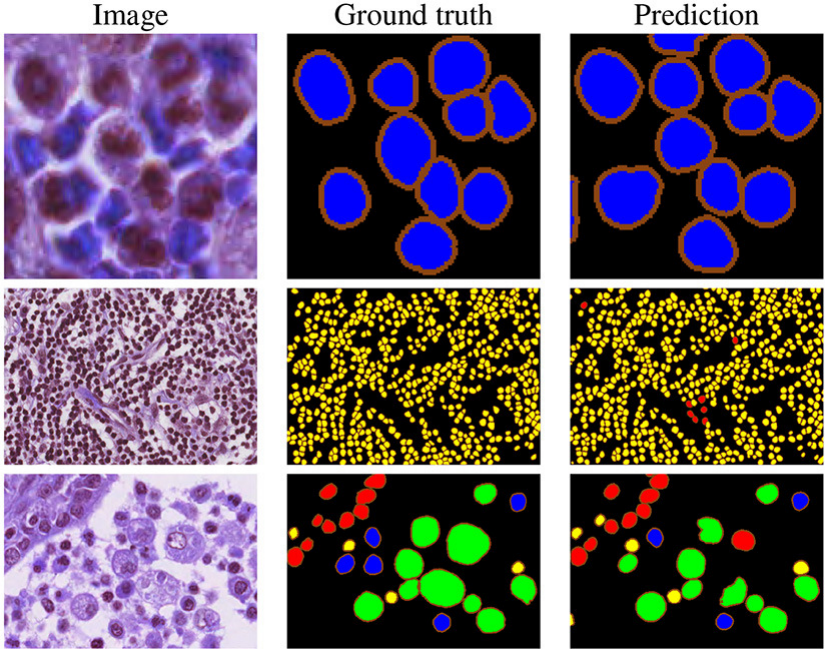
Example results on MoNuSAC images. The colors in the ground truth and segmentation masks represent the different nuclei types (red = epithelial, yellow = lymphocyte, blue = neutrophil, and green = macrophage).

Our proposed method is top-ranked in the MoNuSAC post-challenge leaderboard and would be ranked second considering both legacy and post-challenge phases. For nuclei class-dependent scores, our model achieves the second, second, fourth and second rank for the epithelial, lymphocyte, neutrophil, and macrophage class, respectively. While our method yields very competitive scores in comparison to the top-ranked approach, the results are not directly comparable since the latter used the PanNuke dataset of about 200,000 segmented nuclei (48), i.e., a vastly larger dataset, for pre-training. Samples results from the MoNuSAC experiment are shown in Figure 7.

In general, our results in Tables 2–4 show superior or at least very competitive nuclei instance segmentation performance of our model in comparison to other state-of-the-art methods. Multi-task learning in encoder-decoder-based architectures has become more popular in recent years. Works such as DDU-Net (49) for small-size road detection in high-resolution remote sensing images, ADU-Net (50) for rain and haze removal in natural images or two-stage and dual-decoder convolutional U-Net (51) ensembles for vessel and plaque segmentation in ultrasound images are examples of multi-task models for semantic segmentation or image reconstruction. In our study, we propose a novel dual-task model for a new application, i.e., nuclei instance segmentation in histological images.

While here we report results on datasets that mainly serve for development and benchmarking purposes, our final intention is to make use of our method in either clinical or research applications. Automatic nuclei segmentation and classification are essential tasks in digital pathology; they enable nuclei morphology analysis, cell type classification, as well as cancer detection and grading. Our model can add to the qualitative and quantitative analyses of cells in cancer-affected tissues whenever H&E-stained tissue sections are part of the diagnostic pipeline. For example, in the histopathologic examination of prostate tissue biopsies, nuclei segmentation is still a decisive factor for diagnosing and grading prostate cancer. A concentration of epithelial nuclei on the prostate gland's boundaries indicates an intact tissue structure (the tissue is thus benign). On the other hand, spreading of epithelial nuclei with irregular shapes across the stroma areas suggests that the biopsy sample is malignant (52, 53). Following the detection and segmentation of nuclei with our proposed model for instance segmentation, the classification component of our model could thus be trained to distinguish healthy and malignant nuclei shapes.

Previous work has suggested that the same trained algorithms often yield different performance metrics for tissues from different organs (54, 55). Thus, effective nuclei segmentation methods which can be generalized across various cell, tissue and organ types are required. Our model has demonstrated to perform very well on different datasets containing various organs generated by different laboratories or clinics. Another application scenario is pharmacological research, where imaging technologies have become essential tools for drug development. Here, our method could enable rapid and accurate evaluation of *in vivo* experiments, where the effect of certain drugs on cell number (i.e., nuclei number) or the shape and size of the nuclei should be tested, specifically in organs with a high density of nuclei. If, in this context, it is required to evaluate the effect of the drug on certain cell types, such as immune cells or cancer cells, further training of the classification component of our model might be required.

Last no least, some recent work, such as low-cost U-Net (56) and pruned models (57), introduce computationally less expensive models to reduce inference time and make the CNN-based algorithm more applicable in a real clinical setting, and we aim to extend our work in this direction in our future research.

## 4. Conclusions

Nuclei instance segmentation and classification are essential in analyzing H&E-stained whole slide histological images. In this paper, we have proposed a multi-task encoder-decoder-based model to identify, segment, and if additionally required classify nuclei in histological image patches. The proposed model is demonstrated to yield excellent performance on three benchmark datasets and shown to outperform other state-of-the-art approaches.

## Data availability statement

The datasets presented in this study can be found in online repositories. The names of the repository/repositories and accession number(s) can be found below: https://github.com/masih4/dual_decoder_nuclei_segmentation.

## Ethics statement

Ethical review and approval was not required for the study on human participants in accordance with the local legislation and institutional requirements. Written informed consent for participation was not required for this study in accordance with the National Legislation and the institutional requirements. Ethical review and approval was not required for the animal study because this study was conducted retrospectively using human and animal subject data made available through open access. Ethical approval was not required as confirmed by the license attached with the open access data.

## Author contributions

AM and IE: conceptualization, methodology, and writing–review and editing. GD, RE, and IE: funding acquisition. AM: investigation. IE and SH: supervision. AM, GS, and SH: writing–original draft. All authors read and agreed to the published version of the manuscript.

## Funding

This work was supported by the Austrian Research Promotion Agency (FFG), No. 872636.

## Conflict of interest

Author RE was employed by TissueGnostics GmbH. The remaining authors declare that the research was conducted in the absence of any commercial or financial relationships that could be construed as a potential conflict of interest.

## Publisher's note

All claims expressed in this article are solely those of the authors and do not necessarily represent those of their affiliated organizations, or those of the publisher, the editors and the reviewers. Any product that may be evaluated in this article, or claim that may be made by its manufacturer, is not guaranteed or endorsed by the publisher.

## Footnotes

^1^https://monusac-2020.grand-challenge.org/Results/.

^2^https://monusac-2020.grand-challenge.org/.

^3^https://www.kaggle.com/datasets/ipateam/nuinsseg.

^4^TissueGnostics GmbH https://tissuegnostics.com/products/contextual-image-analysis/strataquest.
